# Load-separation curves for the contact of self-affine rough surfaces

**DOI:** 10.1038/s41598-017-07234-4

**Published:** 2017-07-31

**Authors:** Antonio Papangelo, Norbert Hoffmann, Michele Ciavarella

**Affiliations:** 1 Hamburg University of Technology, Department of Mechanical Engineering, Am Schwarzenberg-Campus 1, 21073 Hamburg, Germany; 2 Imperial College London, Exhibition Road, London, SW7 2AZ UK; 3 Politecnico di BARI. Department of Mechanics, Mathematics and Management, V Gentile 182, 70126 Bari, Italy

## Abstract

There are two main *approximate* theories in the contact of rough solids: Greenwood-Williamson asperity theories (GW) and Persson theories. Neither of them has been fully assessed so far with respect to load-separation curves. Focusing on the most important case of low fractal dimension (D_*f*_ = 2.2) with extensive numerical studies we find that: (i) Persson’s theory describes well the regime of *intermediate* pressures/contact area, but requires significant corrective factors: the latter depend also on upper wavevector cutoff of the roughness; hence, (ii) Persson’s theory does not predict the correct functional dependence on magnification; (iii) asperity theories in the discrete version even neglecting interaction effects are more appropriate in the range of relatively large separations, also to take into consideration of the large scatter in actual realization of the surface.

## Introduction

The closure of rough surfaces has tremendous relevance in a number of scientific and technological applications, from geophysics^1, 2^, to contact electrical and thermal conductance^3, 4^, which under elastic interaction, are also proportional to the contact stiffness due to Barber’s theorem^5, 6^, but also in sealing of valves and lubrication^7^.

The GW model^8^ was a pioneering model for the contact of rough surfaces: starting from a number of assumptions (description of surfaces by *independent* asperities, identical in their radii, with a simple distribution of heights), in the simplest case of an exponential tail of asperity heights leads to a force which is proportional to the number of asperities in contact, and therefore^9^ 13.43–45) a force that is inverse exponential of the mean separation *s* between the nominal mean planes of the surfaces,

1 $$\frac{F(s)}{{E}^{\ast }{A}_{0}}\sim {h}_{s}\exp (\frac{-s}{{h}_{s}})$$

where *E* ^*^ is the plain strain elastic modulus of the contacting materials ($\frac{1}{{E}^{\ast }}=\frac{1-{\nu }_{1}^{2}}{{E}_{1}}+\frac{1-{\nu }_{2}^{2}}{{E}_{2}},$ with *E*_*i*_, *v*_*i*_ respectively the Young modulus and the Poisson ratio) and *h*_*s*_ is a characteristic height of the exponential distribution, which is of the order of the rms amplitude of heights *h*_*rms*_. Using a Gaussian distribution of heights and some results of random process theory, like in the GW–McCool model^10^, the parameters of the GW theory (density of asperities, rms amplitude of summit heights, their density) are obtained in terms of random process theory^11^, and therefore the moments *m*_0_, *m*_2_, *m*_4_ of the Power Spectral Density (PSD) of the rough surface. We recall that the moments *m*_0_, *m*_2_, *m*_4_ are respectively the variance of surface heights, slopes and curvatures. It can be shown that at large dimensionless separation *t* = *s*/*h*_*rms*_, the force decays as ${t}^{-\mathrm{5/2}}\exp (-\,\frac{{t}^{2}}{2})$ (see Supplementary Information), and the result is not much different in more advanced treatment like BGT^12^ which considers the distribution of asperity radii more precisely and found $\frac{F(t)}{{E}^{\ast }{A}_{0}}\sim {t}^{-1}\exp (-\,\frac{{t}^{2}}{2}),$ at large *t*.

A second class of theories on load-separation has been developed by Persson^13^ (notice that, contrary to the case of area-load where Persson’s theory starts from the full-contact, Persson’s load-separation theory assumes low squeezing pressure), assuming from the outset that the area-load relationship is linear and writing the elastic strain energy in partial contact as an empirical correction of the full contact rigorous version, by weighting by the contact area. This leads, for the important case of purely self-affine rough surfaces defined by a power law PSD $C({\bf{q}})={C}_{0}{|{\bf{q}}|}^{-2(1+H)}$ for wavevector ${q}_{0} < |{\bf{q}}| < {q}_{1}$ and zero otherwise, to a force-separation law which assumes the form (ref. 13, eqt. (20) –notice that eq. (20) in ref. 13 was written with the hypothesis *α* = 1).

2 $$\frac{F(s)}{{E}^{\ast }{A}_{0}}\simeq \frac{3}{4}\beta {q}_{0}{h}_{rms}\exp (\frac{-\,\alpha s}{\gamma {h}_{rms}})$$

For the typical case of fractal dimension ${D}_{f}\simeq 2.2$ (where $H=3-{D}_{f}=0.8$ is the Hurst exponent),^13^ suggests the use of $\gamma \simeq 0.4$ from fitting indirect results of ref. 14 (other authors have found $\gamma \simeq 0.48$)^15^, and $\alpha \simeq \mathrm{1,}$ $\beta \simeq \mathrm{1/2}$. Here, the dependence on magnification *ζ* = *q*_1_/*q*_0_ is lost and hence only *q*_0_ is relevant (we shall contradict this qualitatively in our findings). This equation is said to be valid for not too large force so that the area-load relationship is linear–whereas it is not stated there that this has any limit for low forces (as we shall instead find). Notice that^13^ curiously qualitatively seems to return to a form very close to where asperity theories started, an inverse exponential decay (Eq. (1)).

Persson’s theory in the more general case of higher fractal dimensions becomes a lot more complicated, since it predicts a dependence on the upper cutoff of the spectrum *q*_1_ = *ζq*_0_ and involves a dependence on *α*, *β* correcting factors which are given analytically for *γ* = 1, but within the *unknown* approximations. While the area-load relationship of Persson’s theory^16^ has received a number of independent assessments (for the most updated calculation, see ref. 17, and references therein), the load-separation has surprisingly not been studied in detail. Few studies can be found in the scientific literature^18–20^. When looked carefully, Fig. 4 of ref. 18, seems to suggest a remarkable discrepancy of (2) with numerical results (a factor 2 in pressure for a given separation), and the general question on the influence of “finite size” effects on these results has not been assessed. While Gaussian height distribution is often assumed, it is clear that with a fractal self-affine spectrum, we have typically a Fourier series whose terms have different and increasingly smaller “size”, and therefore Central Limit Theorem cannot be relied upon (viceversa, the distribution of slopes and curvature is very easily Gaussian, but this matters less). Also Prodanov *et al*.^19^ found that Persson’s prefactors are about 2–3 times smaller than those found in the simulations (see captions of their Figs 6 and 7), but did not give much importance to them so that it is not clear to the reader how the prefactor should be corrected in future uses. Indeed, Prodanov *et al*.^19^ seem to suggest Persson’s theory gives the correct “functional dependence” in the load-separation curves, while the present investigation clarifies that this is not strictly true, for various reasons: first, a prefactor which changes with magnification hides a “functional dependence” which is not included in the theory, and secondly, Persson’s theory covers the “thermodynamic limit” which can be quite remote from practical surfaces. Sometimes it is suggested that a “roll-off” component of PSD is measured or should be postulated^21^ (whether this is an artifact of the measuring technique would require a separate discussion, see ref. 22). We can obtain the same effect by sticking to pure self-affine power law, and increasing the ratio

3 $$W=\frac{L}{{\lambda }_{0}}$$

between the window size of the problem and the largest wavelength of the power-law spectrum, which increases the number of “nearly equal” components in space and gets us closer to CLT. Ref. 23 discuss how rapidly *W* increases Gaussianity, and similarly^24^. In particular, a ratio *W* = 4 leads to a nice Gaussian bell shape in the central part, but even then, there is a finite limit to the highest peak, and this will have inevitably an effect on the force-separation law. As our technique uses asperities, and there is no problem to match the sides of different surfaces, the window size *W* can be simply increased sticking different surface realizations side-by-side. This leads to PSD that has no power for wavelength greater than *λ*_0_. The *h*_*rms*_ is identical in all realizations.

In this paper, we mainly focus on the most common value of Hurst exponent *H* = 0.8 (i.e. fractal dimension *D*_*f*_ = 2.2) since the lower values are very rare^21, 25^ and we take magnification *ζ* = [32, 64, 128, 256]. We use for the calculations a refined version of a multiasperity code method previously developed^26^, improved for considering periodic boundary conditions. Obviously, the first step is to validate extensively the “multiasperity” code, against various known codes partly also available to the public domain. Since this code is a “multiasperity theory” we find one first perhaps surprising result: while the contact area is affected by the asperity assumption in both the total value and the shape of the contacts, the macroscopic result of the load is not. The only important correction to the classical GW theory is the inclusion of interaction effects, even just to first order, as it is very easy to do^26^.

We find the range of validity of asperity and Persson’s theories, and also some indications of how to use the simplest of the numerical approaches.

## Methods

### Validation of the numerical calculations

The contact code has been developed based on ref. 26: we disregard the modifications made in ref. 27 which were aimed at correcting for the area-load slope, by merging coalescing asperities. A set of rigid summits replace the rough (rigid) substrate which is pressed against an elastic halfspace. The asperities interact to the first order (namely, the effect of each asperity on every other is considered in the asperity center, so that each contact remains Hertzian), and a very simple system of equations is obtained. With respect to ref. 26, the only improvement made is that the loads are self-equilibrated in the window of the problem, i.e. are balanced by a uniform pressure distribution: this obviously corresponds to the periodic b.c. as we verified. In fact, this correction eliminates the mean displacements from the calculation and permits to compute the separation as *s* = *h*_max_ − Δ where *h*_max_ is the maximum asperity height (*h*(*x*) is measured starting from the mean plane of the surface) and Δ is the approach of a remote point.

**Figure 1:**
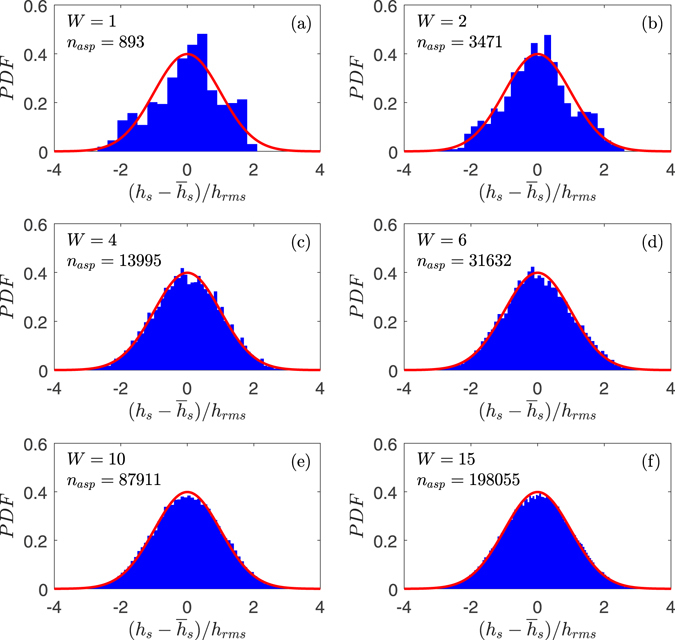
Probability density function of the summit height. The surface has *H* = 0.8, *ζ* = 64, *λ*_0_ = 100 $\mu$m and from (**a**) to (**f**) respectively $W=[1,2,4,6,10,15].$ ${n}_{asp}$ is the number of summits in each realization.

**Figure 2:**
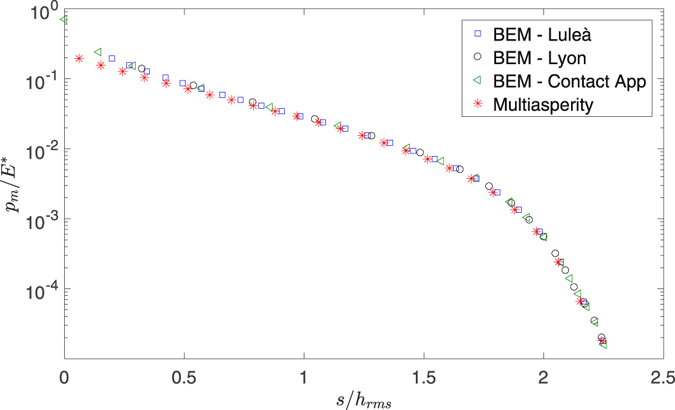
Load vs separation curve for the test surface of Fig. 1a. Red stars symbol indicate the multiasperity code compared with three BEM codes (all with periodic boundary conditions): blue squares from Andreas Almquist, black circles from Philippe Sainsot, green triangles from Lars Pastewka contact-app.

The self-affine surfaces are obtained using a spectral method described in ref. 28. For validation, we first consider a single realization with *H* = 0.8, *ζ* = 64, and window size ratio *W* = 1. We provide the data for the surface in the Supplementary Material in electronic form: actual values *λ*_0_ = 100 *μ*m of the system size and *h*_*rms*_ = 5 *μ*m are not relevant, because of normalizations in the results. We compare the curve load separation obtained with different BEM codes: the first by courtesy of Andreas Almquist based on ref. 29 further refined also to consider periodic boundary conditions, the second is courtesy of Philippe Sainsot of Ecole Centrale de Lyon, and the third is the “contact-app” code realized by Lars Pastewka and available to the public (http://contact.engineering/). This single realization has *n*_*sum*_ = 893 summits when analyzed by the multiasperity code. It deviates significantly from a Gaussian distribution (Fig. 1a), and Fig. 2 shows the dimensionless pressure *p*/*E* ^*^ (where *E* ^*^ is the composite modulus) as a function of the dimensionless separation *s*/*h*_*rms*_: the agreement with all the BEM codes is excellent. We can not make a comparison of CPU time as the two codes did not run on the same computer, but clearly all methods are sufficiently rapid for this kind of problem. Clearly the multiasperity method, when optimized, has potential to be much less time and memory consuming. The comparison with the coalescing asperity model of ref. 27 is not reported, as there is no improvement in load-separation results.

Only at very low separations, the BEM code is needed as the multiasperity results for *s*/*h*_*rms*_ < 0.5 start to be approximate, but this is also the range when Persson’s solution is not valid, as we approach the range close to full contact.

Having validated the code, also with a number of other cases at different magnifications, which we do not report for brevity, we use the multiasperity code only in the following. Increasing the window size *W* the Gaussianity and the number of summits of a single surface improves considerably (Fig. 1b–f).

### Data availability statement

The datasets analysed during the current study are available from the corresponding author on reasonable request.

## Results

### Statistical analysis

**Figure 3:**
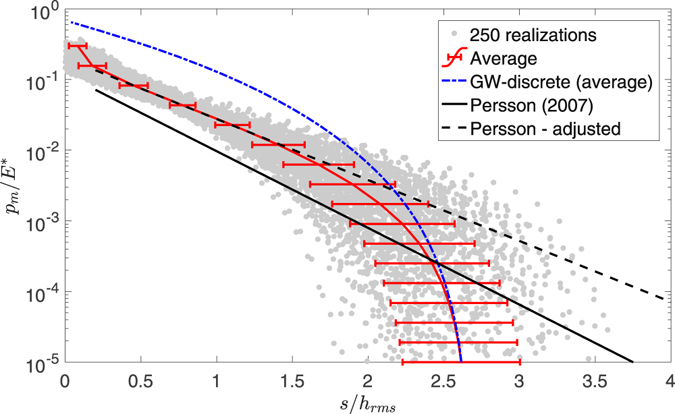
Load vs separation curves (in dimensionless form): single realization curves (gray dots), mean curve over the 250 realizations with error bands indicating one standard deviation (red solid line), Persson model with parameter $\alpha =1,\beta =0.5,\gamma =0.4$ (black solid line, as from ref. 13), Persson model with parameter *α* = 1 $\beta =0.85\pm 0.02,\gamma =0.50\pm 0.01$ (black dashed line, 95% confidence), and the mean curve over 250 surfaces using independent asperities (GW-discrete model, blue dot-dashed line).

In order to obtain statistical relevant results we generate 250 surfaces with *H* = 0.8 *ζ* = 64. The load vs separation curves are plotted in Fig. 3: in particular, gray dots for the result of every single realization, a red solid line for the mean curve over the 250 realizations with error bands indicating one standard deviation, a blue dash-dotted line for the mean over 250 surfaces using a GW-discrete model (not-interacting asperities), a black solid line for the Persson model with parameter $\alpha =1,\beta =\mathrm{0.5,}\gamma =0.4$ (as suggested in ref. 13), a black dashed line for the Persson model with adjusted parameters. Notice that GW-discrete model follows exactly the high separation result of each of the discrete sample realization: computationally, it requires locating summits, simply as local maxima of the surface, taking the geometric mean of the principal radii of curvature, and applying the standard Hertz equation for circular asperities, with a final sum for the macroscopic load. These are incidentally the first steps which we execute anyway for the “multiasperity” code, which additionally considers interaction effects and therefore needs to solve a system of equations, with additional iterations to find the active contact area.

We next compute the fitting parameters of (2) by considering only the part of the curve within $0.5 < s/{h}_{rms} < 1.5$ where the pressure decays exponentially (see Fig. 3) and use eq. (2) with *α* = 1. In fact *α* and *γ* appear together in the exponent thus we had to fit only two parameters (i.e. the scaling factor *β* and the slope *γ*). The result is $\beta =0.85\pm 0.02,\gamma =0.50\pm 0.01$ (provided with 95% of confidence bound) and does not match the the coefficient values provided in ref. 13, with particular remarkable difference in the scaling factor *β*.

Looking at the average curves only, Fig. 3 shows that both the GW-discrete and the Persson model with adjusted parameters are quantitatively in agreement with numerical calculations, but in two different ranges: the GW-discrete model in the high separation limit ($s/{h}_{rms}\approx {h}_{max}/{h}_{rms}$), while the Persson adjusted model in the intermediate separation regime ($s/{h}_{rms}\approx 1$). Obviously, Persson’s model, when used with adjusted fitting parameters (2), is a closed form result, whereas the GW-discrete is a discrete version. But having a particular realization, the use of GW-discrete becomes almost as easy as using a closed form equation, and anyway there is no alternative to obtain an accurate estimate of the load-separation curve in the high separation regime, except for the much more computationally expensive full BEM solution. This should be taken into careful consideration.

### Towards a thermodynamic limit?

**Figure 4:**
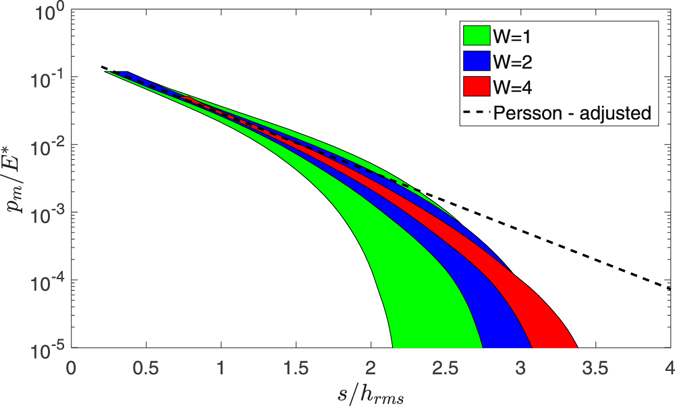
The shaded areas represent load separation curves for *H* = 0.8, *ζ* = 64, and *W* = [1, 2, 4].

Since the finite tails of the Gaussian distribution have such large effect, it is reasonable to argue that a “thermodynamic limit” should eventually occur, although this is really quite an extreme, perhaps unrealistic limit. Indeed, the effect of the window size *W* is studied in Fig. 4. We consider 15 surfaces for each window size (*W* = [1, 2, 4]) and compute the load separation curves. The horizontal width of each shaded area is equal to 2 standard deviations. Increasing the window size, higher summits are obtained and the standard deviation becomes smaller, as each realization is statistically richer (see Fig. 1b–f). Figure 4 shows that *W* mostly affects the load-separation curve in the high separation regime, while in the moderate separation regime the numerical data converge on the Persson adjusted model (dashed line). Therefore, our conclusions about the correction in the Persson prefactor beta is not due to finite-size effects, and are equally valid in the thermodynamic limit.

### Effect of short wavelength cutoff

**Figure 5:**
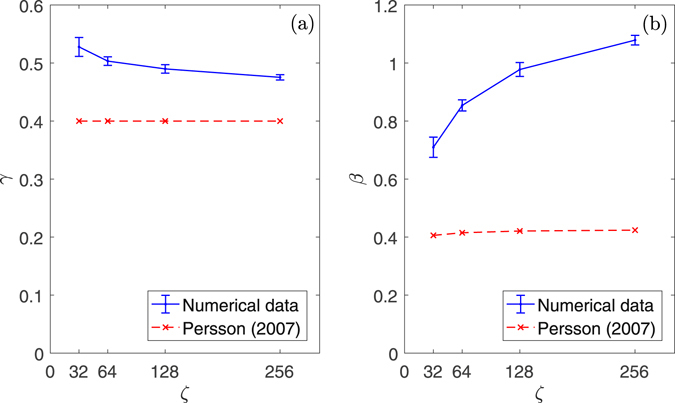
$\gamma =\mathrm{[0.53},0.50,0.49,\mathrm{0.48]}$ and $\beta =\mathrm{[0.71},0.85,0.98,\mathrm{1.08]}$ (respectively (**a**),(**b**)), for *α* = 1, obtained from the fitting of eq. (2) for increasing magnification $\zeta =[32,64,128,256]$ and compared with Persson prediction^13^.

As we have recalled in the introduction, Persson’s^13^ does not predict any effect of the upper truncation wavevector *q*_1_ when the fractal dimension is low. To check this result, in Fig. 5(a,b) we look at the effect of the magnification $\zeta ={q}_{1}/{q}_{0}$ on the parameters *γ* and *β* (error bounds at 95% confidence). We generated 250 rough self-affine surfaces with *H* = 0.8, $\zeta =[32,64,128,256],$ and *W* = 1. We used eq. (2) to fit the results (similar to Fig. 3) in the range where exponential decay is expected ($0.5 < s/{h}_{rms} < 1.5$) and reported *γ* and *β* with 95% confidence error bounds. While the slope parameter *γ* seems to converge to ~0.48 (as suggested by ref. 29) the scaling factor *β* seems to increase with magnification to values much higher than the ones proposed in ref. 13 without showing a clear asymptote. For real surfaces spanning various decades in roughness spectra (and not limited as our computations), the prefactor error in Persson’s equation could reach extremely large values. Some qualitative conclusion of ref. 13 is therefore in error: if Persson’s theory requires corrective prefactors which depend on magnification then it does not predict the correct functional dependence on magnification, and hence the full solution requires more than Persson’s simple scaling. Even assuming we are not interested in the region where finite-size effects apply, one needs to use corrective factors which are given here, and further data would be needed for values outside the range considered, or for surfaces which are not precisely pure power law power spectra.

## Discussion and Conclusion

Persson’s equation is found to be qualitatively similar to the original GW model using exponential height distribution, but the precise fitting parameters in the equation are given here for the first time, for fractal dimension equal to *D*_*f*_ = 2.2 and magnification *ζ* up to 256. We choose the low fractal dimension limit as we aim to provide the useful slope and scaling parameters for the most common fractal dimension of real surfaces. The numerical investigation has been carried on using a multiasperity code which includes interactions among asperities to the first order and has been validated against several BEM codes, proving interaction is the only factor which needs to be taken into account at intermediate separation in asperity theories. We have shown that in the high separation regime Persson’s model greatly overestimate the mean pressure for a given separation, while an adjusted form of the model allows to effectively fit the “intermediate separation” regime ($0.5 < s/{h}_{rms} < 1.5$). In the high separation limit a discrete GW model (with no asperity interactions) is shown instead to converge to the numerical results, and is the only simple option to describe accurately an actual load-separation curve for a particular realization of a surface, in this regime. Both theories are thus valid simply as *fitting equations* but in their specific range of validity. The effect of the ratio between the problem size and the longest wavelength used in the surface generation has been investigated showing that it leads to better Gaussian height distribution and strongly reduces the scatter in the results. Contrary to what suggested by ref. 13, we found that the magnification has an effect even on the low fractal dimension case, in particular on the scaling parameter *β* which increases without showing a clear asymptote. Notice that this was overlooked in the previous works^18–20^ which focused more on the contact stiffness, and when it is plotted against load, only the slope parameter *γ* matters. Finite size effects in contact stiffness were the subject of a heated discussion in the literature a few years ago, see refs 6, 15, 18, 20 and 30. However, the prefactor *β* does not matter if one plots stiffness as a function of load, and hence the results of the present papers are original. Further work is needed to analyze the high fractal dimension case (*D*_*f*_ > 2.5) where Persson theory predicts a more violent influence of the magnification.

## Electronic supplementary material

supplementary informations
