# Supplementary material for: Load-separation curves for the contact of self-affine rough surfaces
Source: Sci Rep. 2017 Jul 31;7:6900. doi: 10.1038/s41598-017-07234-4 (PMC5537336; doi:10.1038/s41598-017-07234-4)
Supplement: Supplementary file 1 — supplementary informations [file 41598_2017_7234_MOESM1_ESM.pdf]

SUPPLEMENTARY INFORMATION

Load-separation curves for the contact of self-affine rough surfaces

Antonio Papangelo[1], Norbert Hoffmann[1,2], Michele Ciavarella[3,*]

[1]Hamburg University of Technology, Department of Mechanical Engineering, Am Schwarzenberg-Campus 1, 21073 Hamburg, Germany
[2]Imperial College London, Exhibition Road, London SW7 2AZ , UK
[3]Polytechnic of BARI. Department of Mechanics, Mathematics and Management, V Gentile 182, 70126 Bari.
[*]corresponding author: m.ciava@poliba.it

1 Appendix

The GW–McCool model [1] obtains the force-separation as follows

$$\frac{F(t)}{A_0} = \frac{4}{3\sqrt{2\pi}} E^* \left(R h_{rms}^3\right)^{1/2} D_{sum} I_{3/2}^g(t) \tag{A.1}$$

where $I_n^g(t) = \int_t^\infty d\xi \, (\xi - t)^n \exp\left(-\xi^2/2\right)$, h_{rms} is RMS of surface heigths (here we are confusing surface "heigths" with "summit heigths"), $t = s/h_{rms}$ is dimensionless separation, R mean radius of curvature, D_{sum} the density of summits, and E^* the plain strain elastic modulus of the contacting materials. From random process theory [2]

$$D_{sum} = \frac{1}{6\pi\sqrt{3}} \frac{m_4}{m_2} \quad ; \quad \frac{1}{R} = \frac{8}{3}\sqrt{\frac{m_4}{\pi}} \quad ; \quad h_{rms} = \sqrt{m_0} \tag{A.2}$$

where m_0, m_2, m_4 are the moments of the PSD, (or else the variance of surface heigths, slopes and curvatures) which depends only on H, ζ

$$\begin{aligned} h_{rms} &= \tfrac{1}{q_0^H}\sqrt{\tfrac{\pi C_0\left(\zeta^{-2H}-1\right)}{H}} \\ \tfrac{1}{R} &= \tfrac{4}{q_0^{H-2}}\sqrt{\tfrac{C_0\left(\zeta^{4-2H}-1\right)}{3\left(q_0^{2H-4}-4\right)}} \\ D_{sum} &= \tfrac{q_0^2(H-1)\left(\zeta^{2(H-1)}-\zeta^2\right)}{8\sqrt{3}\pi(H-2)\left(\zeta^{2(H-1)}-1\right)} \end{aligned} \tag{A.3}$$

Solving the intergal $I^g_{3/2}(t)$ leads to

$$I^g_{3/2}(t) = \frac{1}{2^{3/2}}\sqrt{t}\exp\left(-\frac{t^2}{4}\right)\left[\left(1+t^2\right)\mathrm{K}_{\frac{1}{4}}\left(\frac{t^2}{4}\right) - t^2\mathrm{K}_{\frac{3}{4}}\left(\frac{t^2}{4}\right)\right] \tag{A.4}$$

where $\mathrm{K}_n(x)$ gives the modified Bessel function of the second kind (BesselK[n,z] in Wolfram Mathematica®). In the limit of high separation $t \gg 1$, neglecting higher order terms, eq. (A.4) becomes

$$I^g_{3/2}(t) \simeq \frac{3}{4}\sqrt{\pi}\frac{1}{t^{5/2}}\exp(-\frac{t^2}{4}) \tag{A.5}$$

2 References

[1] J.I. McCool.Comparison of models for the contact of rough surfaces. Wear, 107, 37–60, (1986).

[2] P. R. Nayak. Random process model of rough surfaces. Journal of Tribology, 93(3), 398-407, (1971).
